# Interdisciplinary perspectives on digital technologies for global mental health

**DOI:** 10.1371/journal.pgph.0002867

**Published:** 2024-02-05

**Authors:** Eva Kuhn, Maham Saleem, Thomas Klein, Charlotte Köhler, Daniela C. Fuhr, Sofiia Lahutina, Anna Minarik, Rosemary Musesengwa, Karolin Neubauer, Lotenna Olisaeloka, Francis Osei, Annika Stefanie Reinhold, Ilina Singh, Kerstin Spanhel, Neil Thomas, Tereza Hendl, Philipp Kellmeyer, Kerem Böge

**Affiliations:** 1 Department of Psychiatry and Neurosciences, Campus Benjamin Franklin, Charité –Universitätsmedizin Berlin, Berlin, Germany; 2 Department of Prevention and Evaluation, Leibniz Institute of Prevention Research and Epidemiology-BIPS, Bremen, Germany; 3 Department of Psychiatry and Psychotherapy II, Ulm University, Guenzburg, Germany; 4 Department of Data Science & Decision Support, European University Viadrina, Große, Frankfurt (Oder), Germany; 5 Faculty of Public Health and Policy, Department of Health Services Research and Policy, London School of Hygiene and Tropical Medicine, London, United Kingdom; 6 University of Bremen, Health Sciences, Bremen, Germany; 7 TUM Department of Sport and Health Sciences (TUM SG), Chronobiology and Health, Technical University of Munich, Munich, Germany; 8 TUM Institute for Advanced Study (TUM-IAS), Technical University of Munich, Garching, Germany; 9 Department of Psychology and Neuroscience, Dalhousie University, Halifax, Nova Scotia, Canada; 10 Department of Psychiatry and Welcome Centre for Ethics and Humanities, University of Oxford, Oxford, United Kingdom; 11 Sympatient GmbH, Hamburg, Germany; 12 Institute for Global Health, University College London, London, United Kingdom; 13 Department of Health and Physical Activity, Professorship for Medical Sociology and Psychobiology, University of Potsdam, Potsdam, Germany; 14 Medical Faculty Mannheim, Department of Public Mental Health, Central Institute of Mental Health (CIMH), Heidelberg University, Mannheim, Germany; 15 Faculty of Medicine, Institute for Medical Psychology and Medical Sociology, University of Freiburg, Freiburg im Breisgau, Germany; 16 Centre for Mental Health, Swinburne University of Technology, Hawthorn, Melbourne, Australia; 17 Faculty of Medicine, University of Augsburg, Augsburg, Germany; 18 Institute of Ethics, History and Theory of Medicine, Ludwig-Maximilians-University in Munich, Munich, Germany; 19 Department of Neurosurgery, University of Freiburg—Medical Center, Freiburg im Breisgau, Germany; 20 School of Business Informatics and Mathematics, University of Mannheim, Mannheim, Germany; Tufts University School of Medicine, UNITED STATES

## Abstract

Digital Mental Health Technologies (DMHTs) have the potential to close treatment gaps in settings where mental healthcare is scarce or even inaccessible. For this, DMHTs need to be affordable, evidence-based, justice-oriented, user-friendly, and embedded in a functioning digital infrastructure. This viewpoint discusses areas crucial for future developments of DMHTs. Drawing back on interdisciplinary scholarship, questions of health equity, consumer-, patient- and developer-oriented legislation, and requirements for successful implementation of technologies across the globe are discussed. Economic considerations and policy implications complement these aspects. We discuss the need for cultural adaptation specific to the context of use and point to several benefits as well as pitfalls of DMHTs for research and healthcare provision. Nonetheless, to circumvent technology-driven solutionism, the development and implementation of DMHTs require a holistic, multi-sectoral, and participatory approach.

## Introduction

The potential of digital technologies to foster mental healthcare in underserved communities worldwide is promising, as they are scalable and relatively affordable. Hence, they can potentially close the mental health gap globally [1]. However, structural inequalities and a lack of access to technologies carry the risk that Digital Mental Health Technologies (DMHTs) will primarily serve socio-economically advantaged communities in high-income countries (HIC) [2] that already have better access to healthcare. A multilevel, interdisciplinary, structural, justice-oriented approach is necessary to ensure that DMHTs also benefit underserved, structurally vulnerable communities. Technological interventions are complex and not simply an accessory to in-person clinical mental health services. Currently, the implications of DMHTs in a global context have not been sufficiently explored. This viewpoint builds on emerging interdisciplinary scholarship to better understand the complex innovation dynamics of DMHTs, and their ethical and social implications. We discuss areas crucial to the successful design and implementation of DMHTs which should be considered when addressing the global gap between mental healthcare demand and supply.

## The role of technology in mental health from a global perspective

Mental health can be understood as a highly complex, interactive, multi-layered, and dynamic state that is shaped and influenced by biological, psychological, social, cultural, political, and economic factors. Accordingly, research, prevention, and treatment of mental disorders must move along these dimensions and consider the large variety of determinants of mental health [3].

The global mental health burden has continued to grow into a mental health crisis [4], with a significantly higher treatment gap in low and middle-income countries (LAMICs) than in HICs [5]. A high and diverse number of DMHTs have been shown to be effective in tackling mental health problems—especially during the COVID-19 pandemic—with accelerating digitalization strategies occurring across nations [6]. DMHTs are particularly promising compared to face-to-face treatments as they offer advantages in scalability, accessibility, flexibility, and anonymity [7]. These advantages help to integrate vulnerable people into mental healthcare by reducing potential barriers, namely lack of treatment offers, high travel effort and costs, and the stigmatization of mental illness [8].

Disadvantages of DMHTs include distance from a therapist, less personal social interaction, or spending more time using devices, which may carry its own risks for mental health and wellbeing. Furthermore, DMHTs often fail to address user needs, which can result in the absence of continuous user engagement and high attrition rates in clinical studies [9]. This is particularly true when DMHTs developed in HICs are implemented in LAMICs, but also when targeting diverse populations. In this regard, DMHTs have been shown to be less effective in people with a cultural or ethnic background that differs from the original target group [10]. This may be due to a low fit of DMHTs to differences in particular socio-political conditions and cultural contexts, including different values and approaches to mental health (treatment) concepts as well as population specific health needs [11,12]. With regard to refugee populations, Tekin states that “these chatbots and the method of psychotherapy they endorse impose primarily white and Western standards to mental distress experience and treatment and lack the sensitivity to different forms of experiences” [13]. In some cases, to address such social and cultural differences and, herewith, adjust the fit of DMHTs, it seems appropriate to integrate the cultural values and context of the target group by culturally adapting treatments [14]. For example, DMHTs have been culturally adapted to include differences on a surface level, such as simplified navigation in apps or the adaptation of example characters and illustrations. On a deeper level, DMHTs have also been adapted to reflect on multilevel burdens, a potential stigmatization of mental disorders or the relevance of religion. However, cultural adaptations of DMHTs are often overlooked, poorly-reported, or not standardized [15,16]. Thus, although an overall influence of cultural adaptation of DMHTs on their effectiveness is suggested, it is not yet known which adaptation to which cultural differences is essential for the acceptance or effectiveness of DMHTs. In other cases, in which adaptation has not been deemed preferable and appropriate, such as in contexts involving decolonizing populations, experts have argued for the crucial importance of community-led and sovereign design, centering the health needs, agency, but also ontologies and epistemologies of oppressed user communities as necessary prerequisites to recovery [17].

## Digital mental health technology

**Fig 1:**
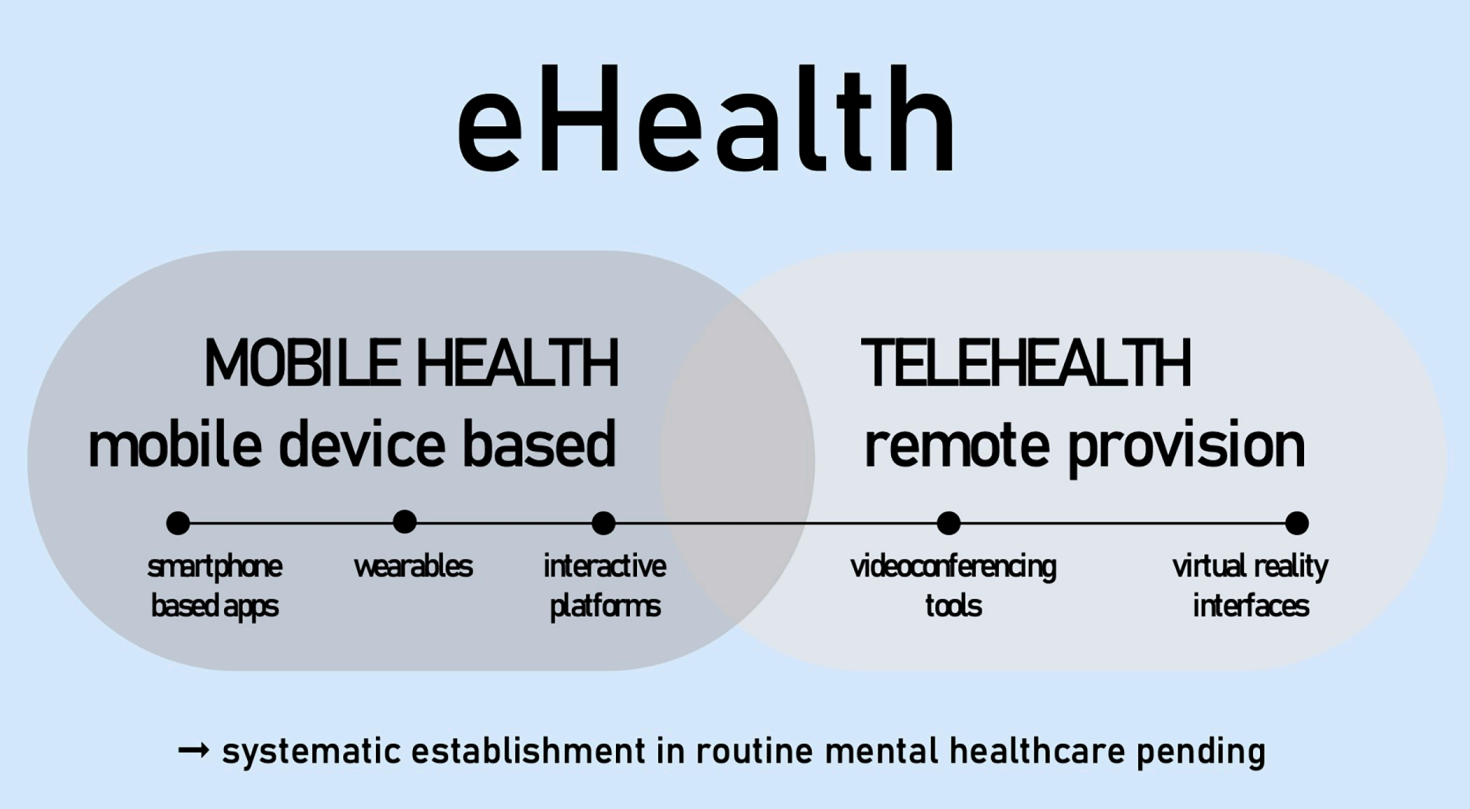
Categorisation of digital mental health technology.

As a viable and user-elected option in a particular socio-political context, cultural adaptation should be factored in from the very beginning of the development and implementation of DMHTs. Depending on the context, the technology used can range from SMS- or website-based interventions over videoconferencing (‘telehealth’) to smartphone- or wearable-based apps (see Fig 1).

Multimodal data analytics, including adaptive machine learning (especially deep neural networks) makes it possible to integrate data from different sources such as electronic health records, wearables, and smartphones that enable ‘deep digital phenotyping’ (i.e., identifying and relating digital patterns to specific states of health or disease) [18]. Another potential of DMHTs lies in the possibility of collecting momentary, daily life data and generating rich longitudinal information on the outcomes and influencing factors of mental health. Ecological momentary assessment (EMA) is well-suited for collecting such dynamic time-series data. EMA uses repeated, real-time assessments several times daily, following a time-based, event-based, or a combined sampling design. In this regard, one important distinction is made between ‘passive’ and ‘active’ data. Passive data refers to data streams that are collected without active user input, such as data on a person’s location or from specific sensors that measure physiological or physical-environmental data. In contrast, active data typically require user actions such as ratings or natural language input. This information can then be harnessed to deliver an ecological momentary intervention (EMI), allowing for time- and context-specific targeting of individual needs [7]. From a global perspective, EMAs and EMIs can help to develop a more granular and context-specific understanding of the interrelationships between different social determinants of mental health and their fluctuations. This can then form the basis for early and preventive interventions and enhance personalized healthcare. EMIs can also include factors such as participatory design and research, feedback options within the application, ease of use, gamification, personalization options, symptom monitoring, app socialization, and integration with other health services [9]. As these types of applications can collect a large variety of data, from mood to geographical or social interaction data, they foster a multi-perspective view on mental health and thereby collaboration across different disciplines. An interdisciplinary research approach including, for example, regulatory, computational, or design input, next to a clinical perspective, is further required to meet the different stakeholder needs. This is particularly important to increase engagement and address user needs in their daily lives.

With regards to machine learning in general, critical voices have argued that not all participatory approaches in the design of a DMHT are appropriate to scale these technologies [19]. It is therefore crucial that co-designs and involvement of all relevant stakeholders is sought throughout the life and research cycle, i.e. long-term, that the specific context of participation is taken into account and that users’ participation is recognized as work they are compensated for. Moreover, aspects that can be quantified as easily as, for example, vital signs, should also be considered in algorithmic prediction and particularly when basing treatment decisions on such a prediction. Among such aspects are a patient’s values, their attitude towards life or beliefs [6,20].

To realize the full potential of DMHTs in research and practice, certain structural-technological requirements need to be met (i.e., they should be embedded in a functioning digital infrastructure). This can involve stable energy supplies as well as steady and fast internet connectivity, as most functions are mediated via server-based technologies. In countries with a well-established national approach to DMHTs, such as Australia and the UK, web-based platforms are used as the primary delivery modality, most commonly for the provision of psycho-educational material, self-guided cognitive behavioral therapy programs, and peer-to-peer forums [21]. This has enabled wide access to mental health information and treatment that meet community needs at scale, often as part of a stepwise care framework. However, these conditions are not necessarily met worldwide. In countries with an emerging digital infrastructure, DMHTs are not readily available and a persistent digital divide will likely pose substantial obstacles to DMHT research and implementation [22].

## Economics

Further factors that influence technological development and implementation involve economic considerations. Economic models can be used to assess the value of health interventions by analyzing the relationship between health outcomes and costs incurred and then comparing them to existing alternatives [23]. A long-term assessment of health outcomes is needed to evaluate the impact of interventions for mental disorders. Frequently, the quality-adjusted life year (QALY) is used to measure patients’ quality of life as a function of the severity of symptoms over a period of several years. The incurred costs of an intervention consist of direct and indirect costs such as the costs of developing the intervention and its components, or the costs to society [24]. An important aspect to consider when creating DMHTs is the differences in their validity and applicability in different socio-political contexts and cultures. Verifying the benefit of digital tools within these models should consider the speed of regulation, funding tools, development, and the quality of the tools developed. Although challenging, creating models to assess the value of DMHTs requires parameterization to the econometric context of the underlying healthcare system within a country.

When considering the global implementation of DMHT, it is important to consider various factors, namely:

- Cross-cultural features and stigma. In mental illness, aside from measuring the effectiveness of an intervention (which is challenging to quantify accurately, see Jankovic et al. [25]), there are cross-cultural dynamics and stigma that greatly affect patients’ quality of life. Thus, an all-encompassing view of assessing patients’ cultural and disorder-related health is a major challenge.
- Availability and adoption of digital tools. The existence of available digital tools has an impact on the prospect of using a digital intervention [26]. However, the risk of poor adoption of digital tools and the related effect on value is not inherent in cost modeling and requires separate sensitivity and scenario analyses. Additionally, there is not always a clear correlation between the economic situation and digital adoption rate per country. For example, in Ukraine and Libya, both LAMICs, more than 63% versus 18% of the population use the internet [27].
- Validity assessment. Validity of the DMHT involves checking the effectiveness of the instrument, its psychometric validity, and country-specific adaptation. Language, cultural and/or socio-political differences as well as the agency of user communities significantly affect the process of assessing the validity of a DMHT. These factors call attention to the cross-cultural implementation of DMHTs as a separate and very important process, which is worth paying attention to.
- Resource inequality. There is a large difference between HICs and LAMICs in resources made available for the diagnosis and treatment of mental disorders. LAMICs often fall well below the average of 3.96 psychiatrists available per 100,000 population [28]. Hence, especially in countries where resources for mental disorders are still scarce, no valid benchmark can be found against which the benefit of digital interventions can be compared.

## Ethics

In line with these disparities, the Lancet Commission on Global Mental Health emphasized the “vast inequities (…) in the distribution of mental health resources, not only between but also within countries” [5]. While there is widespread hope that DMHTs may be able to address some of these injustices, they need to be oriented towards health justice to materialize this potential and foster mental health as a public health good. This is even more pressing as structural and intersectional concerns regarding justice have not been emphasized in (Western) ethics. Currently, more individualistic issues of data privacy, informed consent, or autonomy have been debated, neglecting globally pressing concerns of equitable health outcomes [29].

The steering of DMHTs towards health justice is crucial for the facilitation of population-wide and global health benefits and the strengthening of health equity. Working towards these aims will require greater accounting for and addressing of socio-political determinants of health, instead of predominant responsibilization of the individual for health outcomes and self-management via DMHTs [30]. Mental health is fundamentally shaped by social, cultural, and political factors that affect human environments, including structural racism and intergenerational trauma from the ongoing legacy of colonialism [31]. Prioritizing the health needs of systematically oppressed and under-served communities is warranted and will require the de-centering of Western approaches to health and healthcare and the centering of excluded and structurally vulnerable communities, their agency, health needs, and expertise [1,2,29].

To foster the development of socially sustainable and globally beneficent DMHTs, participatory research and co-creation have been proposed as vital steps. Some have emphasized the need for conducting qualitative research with target groups at various stages of intervention development and cultural adaptation [14]. Yet, to eliminate issues of commodification, re-presentation, paternalization, and (data) colonialism that have shaped biomedical research, medicine, and healthcare, under-served communities need greater power over DMHT interventions at all stages. Some of these populations, such as Indigenous people, have requested sovereignty over their data, governance, and ownership of digital technologies as a necessity to the improvement of their health and wellbeing [29]. Further issues regarding access to DMHTs need to be addressed as the global shift towards greater reliance on DMHTs risks amplifying the digital divide [32].

## Global health policy and governance

Translating laws into application codes remains a difficulty that requires constant communication with interest groups involved in providing DMHTs. Mandatory standards for quality assurance are insufficient in both high- and low-income settings. Due to their complexity, DMHTs require granular and context-sensitive regulatory and policy frameworks to promote effective and just organizational and clinical governance structures that establish, sustain, and improve the integration of DMHTs into the broader healthcare system [33].

DMHTs need to be integrated into complex health systems that are more like networks of care nodes. Complex health systems and their resistance to change present numerous risks to DMHT’s stability and sustainability. This holds particularly true for countries in the global North, where legacy systems and regulatory frameworks mandate extensive reconstruction or retrofitting to effectively assimilate emerging digital frameworks [34]. In contrast, emerging economies, many of which lack established digital health policies and frameworks, face fewer of these challenges. This grants them more flexibility to integrate new digital technologies right from the onset in a resource-effective manner [34]. On the downside, the absence of robust regulatory frameworks could also abet data mining as well as the implementation of unsafe technologies. Hence, the WHO’s digital health strategy emphasizes leveraging digital technology to improve service delivery, health information systems, and health workforce development for sustainable and equitable health systems. The four guiding principles for sustainable adoption of digital health technology in health policies are commitment, maximum integration in the health system, acceptable use, and addressing impediments [35].

To foster successful and sustainable integration of DMHTs into a healthcare system, a robust digital infrastructure is necessary. DMHTs should provide equitable solutions and not widen current disparities. Especially in LAMICs, policymakers and stakeholders may take advantage of a leapfrog approach. Leapfrogging means to bypass certain stages of (technological) development such as the landline phones era and directly introduce smartphones. This may result in a significant improvement of (mental) health services and their delivery. Nonetheless, issues such as limited resources or socio-cultural viability of technologies in particular contexts have to be taken into account [36]. In order to address these issues, it is required to strengthen the commitment and systematic engagement of all stakeholders to build human and institutional capacity for the safe and contextually appropriate use and scale-up of DMHTs (for a mapping of stakeholders in Global Mental Health in LAMICs, see [37]). To maximize the impact of DMHTs globally, digital health literacy needs to be improved in HICs and LAMICs equally. By fostering stakeholder engagement between public and private health programs, policymakers can address this and inform the public. In addition, the curriculum of mental health training should include DMHTs and the ethical issues it raises to promote digital health literacy at all levels [38].

## Legal aspects and regulation

Due to the pace at which DMHTs are developed, countries most often lag behind with legislation. In an attempt to bridge this law-technology gap, existing legal frameworks have been applied to embed DMHTs into national and international law. Among these frameworks, effectiveness and safety legislation classify DMHTs as medical devices, e.g., the Medical Device Regulation in the European Union. In a similar vein, legislation of the US Food and Drug Administration provides non-binding guidelines concerning the effectiveness of new healthcare technologies, leaving space for stricter laws, especially for mental health apps that often lack detailed regulation, or for responsible sectors that are isolated from each other [39].

Another area of legislation applicable to DMHTs concerns consumer protection laws. In light of growing evidence of data-mining approaches, strict laws on medical and health data protection are essential to ensure lawful processing that prevents the criminal misuse of biomedical data [40]. Due to expected discrimination and stigmatization of people with mental disorders, mental health data are subject to serious privacy and confidentiality concerns [41].

In a response to legislative activity and in order to forestall its intensification, commercial stakeholders are increasingly adopting compliance via by-design approaches (e.g., ethics-by-design) that seek to avoid legal pitfalls during the design phase of DMHTs. Such approaches promote an alignment between industry and jurisdiction at an early stage. Despite such positive developments, legislation on DMHTs face numerous challenges such as asymmetries between providers and consumers of mental health apps. As these challenges transcend national borders, they require harmonization of DMHT jurisdiction on an international level. Although not justiciable, using human rights as the legal framework applied to DMHT development and deployment allows for inclusive and equitable legislation. Among those at highest risk of being denied the fundamental human right to (mental) healthcare, there are also those whose human rights are already at risk for reasons of poverty, migration, and socio-historical injustices.

Nevertheless, legal frameworks, especially at the level of international human rights law (e.g., UNESCO soft law) and humanitarian law and their respective regional and national laws, only provide basic legal protection as an important prerequisite to promote equity and justice. Hence, these basic legal protections need to be complemented by a wide range of additional multi-level initiatives, tools and interventions to promote an understanding and implementation of justice and equity in global mental health that goes beyond breaking laws. At the level of international organizations, one example is provided by the World Health Organization’s (WHO) recent initiative on universal health coverage for mental health [42]. At the level of countries and local communities, there is now a wide range of digital mental health initiatives with a focus on access and other aspects of justice and equity in LAMICs. One example, specifically in the area of ethics of digital mental health is the Ethics for Mental Health Digital Innovation for Young People in Africa (EMDIYA) Network. One remaining challenge is to translate these flourishing initiatives and actions into health policy interventions that ensure sustainable, effective and intersectionally beneficent improvements in mental health justice and equity.

## Conclusion

DMHTs have significant potential for global mental health. Their technological development raises hope of easing economic burdens and could also bridge issues of equity if they are grounded in and oriented towards health justice. Prioritizing and empowering underserved populations as agents of DMHTs and their cultural adaptations is crucial for fostering socially sustainable and globally beneficent DMHTs with equitable health outcomes [14,29].

The development, evaluation, and implementation of effective DMHTs remains complex and a more evidence-based and balanced understanding of their merits and drawbacks is needed. DMHTs that operate without links to the healthcare system involve the risk of sliding into technology-driven solutionism with a truncated understanding of (mental) health. This issue warrants a deliberate and holistic approach to DMHTs, their socially and ethically justifiable design, as well as regulation and policy action that will support multi-sectoral cooperation, community participation, and infrastructure. To strengthen the development and implementation of feasible, acceptable, and effective DMHTs, the collaboration of stakeholders at a cross-cultural and global level is needed that prominently centers the needs and agency of under-served communities. Only if all the areas raised are integrated, DMHTs will become vital for healthcare and global mental health.

## References

[1] Adepoju P. Africa turns to telemedicine to close mental health gap. Lancet Digit Health 2020;2:e571–e572. doi: 10.1016/S2589-7500(20)30252-1 33103096 PMC7571965

[2] Herzog L, Kellmeyer P, Wild V. Digital behavioral technology, vulnerability and justice: towards an integrated approach. Review of Social Economy 2022;80:7–28.

[3] Alegría M, NeMoyer A, Falgàs Bagué I, Wang Y, Alvarez K. Social Determinants of Mental Health: Where We Are and Where We Need to Go. Curr Psychiatry Rep 2018;20:95. doi: 10.1007/s11920-018-0969-9 30221308 PMC6181118

[4] GBD 2019 Mental Disorders Collaborators. Global, regional, and national burden of 12 mental disorders in 204 countries and territories, 1990–2019: a systematic analysis for the Global Burden of Disease Study 2019. Lancet Psychiatry 2022;9:137–50. doi: 10.1016/S2215-0366(21)00395-3 35026139 PMC8776563

[5] Patel V. Mental health in low- and middle-income countries. Br Med Bull 2007;81–82:81–96. doi: 10.1093/bmb/ldm010 17470476

[6] The LDH. Mental health in the digital age. Lancet Digit Health 2022;4:e765. doi: 10.1016/S2589-7500(22)00191-1 36229344

[7] Balaskas A, Schueller SM, Cox A. L, Doherty G. Ecological momentary interventions for mental health: A scoping review. PLoS One 2021;16:e0248152. doi: 10.1371/journal.pone.0248152 33705457 PMC7951936

[8] Byrow Y, Pajak R, Specker P, Nickerson A. Perceptions of mental health and perceived barriers to mental health help-seeking amongst refugees: A systematic review. Clin Psychol Rev 2020;75:101812. doi: 10.1016/j.cpr.2019.101812 31901882

[9] Nwosu A, Boardman S, Husain MM, Doraiswamy P. M. Digital therapeutics for mental health: Is attrition the Achilles heel? Front Psychiatry 2022;13:900615. doi: 10.3389/fpsyt.2022.900615 35982936 PMC9380224

[10] Karyotaki E, Ebert DD, Donkin L, Riper H, Twisk J, Burger S, et al. Do guided internet-based interventions result in clinically relevant changes for patients with depression? An individual participant data meta-analysis. Clin Psychol Rev 2018;63:80–92. doi: 10.1016/j.cpr.2018.06.007 29940401

[11] Connelly K, Stein KF, Chaudry B, Trabold N. Development of an Ecological Momentary Assessment Mobile App for a Low-Literacy, Mexican American Population to Collect Disordered Eating Behaviors. JMIR Public Health Surveill 2016;2:e31. doi: 10.2196/publichealth.5511 27418020 PMC4965614

[12] Heim E, Kohrt BA. Cultural Adaptation of Scalable Psychological Interventions: A New Conceptual Framework. CPE 2019;1.

[13] Tekin Ş. Is Big Data the New Stethoscope? Perils of Digital Phenotyping to Address Mental Illness. Philos. Technol. 2021;34:447–61.

[14] Spanhel K, Balci S, Feldhahn F, Bengel J, Baumeister H, Sander LB. Cultural adaptation of internet- and mobile-based interventions for mental disorders: a systematic review. NPJ Digit Med 2021;4:128. doi: 10.1038/s41746-021-00498-1 34433875 PMC8387403

[15] Aubel J, Chibanda D. The neglect of culture in global health research and practice. BMJ Glob Health 2022;7:e009914.

[16] Montenegro CR, Ortega F. Thinking beyond implementation: context and culture in global mental health. BMJ Glob Health 2020;5(12). doi: 10.1136/bmjgh-2020-004539 33380416 PMC7780541

[17] Asher BlackDeer A. Culture as Treatment: A Pathway toward Indigenous Health Equity. hpj 2023;3.

[18] Birk RH, Samuel G. Digital Phenotyping for Mental Health: Reviewing the Challenges of Using Data to Monitor and Predict Mental Health Problems. Curr Psychiatry Rep 2022;24:523–28. doi: 10.1007/s11920-022-01358-9 36001220

[19] Sloane M, Moss E, Awomolo O, Forlano L. Participation Is not a Design Fix for Machine Learning. In: Equity and Access in Algorithms, Mechanisms, and Optimization (EAAMO ‘22). doi: 10.1145/3551624.3555285

[20] Tsamados A, Aggarwal N, Cowls J, Morley J, Roberts H, Taddeo M, et al. The ethics of algorithms: key problems and solutions. AI & Soc 2022;37:215–30.

[21] Cross SP, Karin E, Staples LG, Bisby MA, Ryan K, Duke G, et al. Factors associated with treatment uptake, completion, and subsequent symptom improvement in a national digital mental health service. Internet Interv 2022;27:100506. doi: 10.1016/j.invent.2022.100506 35242587 PMC8857488

[22] Kozelka EE, Jenkins JH, Carpenter-Song E. Advancing Health Equity in Digital Mental Health: Lessons From Medical Anthropology for Global Mental Health. JMIR Ment Health 2021;8:e28555. doi: 10.2196/28555 34398788 PMC8406126

[23] Gega L, Jankovic D, Saramago P, Marshall D, Dawson S, Brabyn S, et al. Digital interventions in mental health: evidence syntheses and economic modelling. Health Technol Assess 2022;26:1–182. doi: 10.3310/RCTI6942 35048909 PMC8958412

[24] Mitchell LM, Joshi U, Patel V, Lu C, Naslund JA. Economic Evaluations of Internet-Based Psychological Interventions for Anxiety Disorders and Depression: A Systematic Review. J Affect Disord 2021;284:157–82. doi: 10.1016/j.jad.2021.01.092 33601245 PMC8008508

[25] Jankovic D, Bojke L, Marshall D, Saramago Goncalves P, Churchill R, Melton H, et al. Systematic Review and Critique of Methods for Economic Evaluation of Digital Mental Health Interventions. Appl Health Econ Health Policy 2021;19:17–27. doi: 10.1007/s40258-020-00607-3 32803521

[26] Sieck CJ, Sheon A, Ancker JS, Castek J, Callahan B, Siefer A.Digital inclusion as a social determinant of health. NPJ Digit Med 2021;4:52. doi: 10.1038/s41746-021-00413-8 33731887 PMC7969595

[27] Kepios. DataReportal. 2022. [Cited 2022 November 23]. Available from: https://datareportal.com/.

[28] Rathod S, Pinninti N, Irfan M, Gorczynski P, Rathod P, Gega L, et al. Mental Health Service Provision in Low- and Middle-Income Countries. Health Serv Insights 2017;10. doi: 10.1177/1178632917694350 28469456 PMC5398308

[29] Hendl T, Roxanne T. Digital surveillance in a pandemic response: What bioethics ought to learn from Indigenous perspectives. Bioethics 2022;36:305–12. doi: 10.1111/bioe.13013 35180324

[30] Martinez-Martin N, Dasgupta I, Carter A, Chandler JA, Kellmeyer P, Kreitmair K, et al. Ethics of Digital Mental Health During COVID-19: Crisis and Opportunities. JMIR Ment Health 2020;7:e23776. doi: 10.2196/23776 33156811 PMC7758081

[31] Wilk P, Maltby A, Cooke M. Residential schools and the effects on Indigenous health and well-being in Canada-a scoping review. Public Health Rev 2017;38:8. doi: 10.1186/s40985-017-0055-6 29450080 PMC5809999

[32] Makri A. Bridging the digital divide in health care. The Lancet Digital Health 2019;1:e204–e205.

[33] Goldman ML, Druss BG, Horvitz-Lennon M, et al. Mental Health Policy in the Era of COVID-19. Psychiatr Serv 2020;71:1158–62. doi: 10.1176/appi.ps.202000219 32517639

[34] World Economic Forum. Health Systems Leapfrogging in Emerging Economies: Project Paper; 2014. [Cited 2023 September 16]. Available from: https://www3.weforum.org/docs/WEF_HealthSystem_LeapfroggingEmergingEconomies_ProjectPaper_2014.pdf.

[35] World Health Organization. Global strategy on digital health 2020–2025; 2021. License: CC BY-NC-SA 3.0 IGO.

[36] Oduor K, Ogweno S, Ajwang D, Okinyi N. Incorporating mHealth Interventions into Kenya’s Health Infrastructure to Augment Universal Health Coverage, Service Delivery Improvement Approach. SEEJPH 2023. doi: 10.56801/seejph.vi.209

[37] Iemmi V. Sustainable development for global mental health: a typology and systematic evidence mapping of external actors in low-income and middle-income countries. BMJ Glob Health 2019;4:e001826. doi: 10.1136/bmjgh-2019-001826 31908860 PMC6936513

[38] Akbarialiabad H, Bastani B, Taghrir MH, Paydar S, Ghahramani N, Kumar M. Threats to Global Mental Health From Unregulated Digital Phenotyping and Neuromarketing: Recommendations for COVID-19 Era and Beyond. Front Psychiatry 2021;12:713987. doi: 10.3389/fpsyt.2021.713987 34594251 PMC8477163

[39] Parker L, Bero L, Gillies D, Raven M, Grundy Q. The “Hot Potato” of Mental Health App Regulation: A Critical Case Study of the Australian Policy Arena. Int J Health Policy Manag 2019;8:168–76. doi: 10.15171/ijhpm.2018.117 30980633 PMC6462196

[40] Tikkinen-Piri C, Rohunen A, Markkula J. EU General Data Protection Regulation: Changes and implications for personal data collecting companies. Computer Law & Security Review 2018;34:134–53.

[41] Lustgarten SD, Garrison YL, Sinnard MT, Flynn AWP. Digital privacy in mental healthcare: current issues and recommendations for technology use. Curr Opin Psychol 2020;36:25–31. doi: 10.1016/j.copsyc.2020.03.012 32361651 PMC7195295

[42] World Health Organization. The WHO special initiative for mental health (‎2019–2023)‎: universal health coverage for mental health; 2019. License: CC BY-NC-SA 3.0 IGO.

